# Incidence and risk factors for venous thromboembolism in the Cancer-VTE Registry stomach cancer subcohort

**DOI:** 10.1007/s10120-023-01378-1

**Published:** 2023-04-01

**Authors:** Takaki Yoshikawa, Takeshi Sano, Masanori Terashima, Kensei Yamaguchi, Etsuro Bando, Ryohei Kawabata, Hiroshi Yabusaki, Hisashi Shinohara, Mari S. Oba, Tetsuya Kimura, Atsushi Takita, Mitsuru Sasako

**Affiliations:** 1 Department of Gastric Surgery, National Cancer Center Hospital, 5-1-1 Tsukiji, Chuo-ku, Tokyo, 104-0045 Japan; 2 Department of Gastroenterological Surgery, Cancer Institute Hospital of the Japanese Foundation for Cancer Research, Tokyo, Japan; 3 Division of Gastric Surgery, Shizuoka Cancer Center, Shizuoka, Japan; 4 Department of Gastroenterological Chemotherapy, Cancer Institute Hospital of the Japanese Foundation for Cancer Research, Tokyo, Japan; 5 Department of Surgery, Osaka Rosai Hospital, Sakai, Japan; 6 Department of Surgery, Sakai City Medical Center, Sakai, Japan; 7 Department of Gastroenterological Surgery, Niigata Cancer Center Hospital, Niigata, Japan; 8 Department of Gastroenterological Surgery, Division of Upper GI, Hyogo Medical University, Nishinomiya, Japan; 9 Department of Medical Statistics, Toho University, Tokyo, Japan; 10 Department of Clinical Data Science, Clinical Research and Education Promotion Division, National Center of Neurology and Psychiatry, Tokyo, Japan; 11 Primary Medical Science Department, Daiichi Sankyo Co., Ltd, Tokyo, Japan; 12 Data Intelligence Department, Daiichi Sankyo Co., Ltd, Tokyo, Japan; 13 Department of Surgery, Yodogawa Christian Hospital, Osaka, Japan

**Keywords:** Stomach cancer, Venous thromboembolism, Japan

## Abstract

**Background:**

The Cancer-VTE Registry was a large-scale, multicenter, prospective registry designed to investigate real-world data on venous thromboembolism (VTE) incidence and risk factors in adult Japanese patients with solid tumors. This pre-specified subgroup analysis aimed to estimate the incidence of VTE, including VTE types other than symptomatic VTE, and identify risk factors of VTE in stomach cancer from the Cancer-VTE Registry.

**Methods:**

Stage II–IV stomach cancer patients who planned to initiate cancer therapy and underwent VTE screening within 2 months before registration were enrolled.

**Results:**

Of 1,896 patients enrolled, 131 (6.9%) had VTE at baseline, but 96.2% were asymptomatic. Female sex, age ≥ 65 years, VTE history, and D-dimer > 1.2 μg/mL were independent risk factors of VTE at baseline. Notably, patients with D-dimer > 1.2 µg/mL at the time of cancer diagnosis had an approximately 20-fold risk of VTE. During follow-up, event incidences were symptomatic VTE, 0.3%; incidental VTE requiring treatment, 1.1%; composite VTE, 1.4%; bleeding, 1.6%; cerebral infarction/transient ischemic attack/systemic embolic events, 0.7%; and all-cause death, 15.0%. The incidence of all-cause death was higher in patients with VTE vs without VTE at baseline (adjusted hazard ratio 1.67; 95% confidence interval 1.21–2.32; p = 0.002).

**Conclusions:**

VTE prevalence at the time of cancer diagnosis was not negligible and was extremely high when the patients had high D-dimer. VTE screening by D-dimer before starting cancer treatment is advisable, even for asymptomatic patients, regardless of whether the patient is undergoing surgery or chemotherapy.

**Trial registration:**

UMIN000024942.

**Supplementary Information:**

The online version contains supplementary material available at 10.1007/s10120-023-01378-1.

## Introduction

Cancer patients have at least a four-fold higher incidence of venous thromboembolism (VTE) compared with patients without cancer [1–3]. Several factors, such as age, cancer site, stage, and time after the cancer diagnosis, affect the incidence of VTE [2, 4]. The incidence of VTE is known to be significantly influenced by ethnicity and is reportedly lower in Asian compared with Western patients [5–7]. Nevertheless, the incidence of VTE in Japanese populations has been increasing recently [8], mainly because of improvements in diagnostic techniques and increased frequency of screening, highlighting the risk of VTE even among Asian patients with cancer.

Stomach cancer is the fifth leading cause of death among all neoplasms worldwide in 2022 [9]. Moreover, the prevalence of stomach cancer is high in East Asian countries [9]. Based on the Khorana, Vienna CATS, PROTECHT, and CONKO VTE risk assessment tools, stomach cancer has the highest risk of VTE among all neoplasms [4].

Previously, most Asian studies on VTE and stomach cancer focused on specific patient background characteristics, such as only surgical patients or only patients with advanced cancer requiring chemotherapy. Moreover, most reports cover single-center retrospective studies. Thus, reliable, comprehensive data on the risk of VTE in Asian populations are limited for various types of cancer treatment.

The Cancer-VTE Registry, which was a large-scale, multicenter, prospective registry in Japanese patients with solid tumors, including colorectal, lung, stomach, pancreatic, breast, and gynecologic cancer, estimated the prevalence of VTE before initiating cancer treatment and the incidences of symptomatic VTE, bleeding, and all-cause death during a 1-year follow-up [10–12]. In the baseline and 1-year follow-up reports of the Cancer-VTE Registry, the prevalence of VTE before initiating cancer treatment was an independent risk factor for symptomatic VTE, bleeding events, and all-cause death during subsequent treatment. Additionally, patients with stomach cancer had the second highest prevalence of VTE (6.9%) among the six types of solid tumors, of whom 0.3% of patients had symptomatic VTE during the 1-year follow-up [12]. However, no further detailed data have been reported on patients with stomach cancer. The objective of this pre-specified subgroup analysis was to estimate the incidence of VTE, including VTE types other than symptomatic VTE, and identify risk factors of VTE in Japanese patients with stomach cancer from the Cancer-VTE Registry.

## Materials and methods

### Study design

The rationale and design of the Cancer-VTE Registry have been published previously [10]. The Cancer-VTE Registry was a nationwide clinical registry conducted between March 2017 and February 2019, with a 1-year follow-up that ended in February 2020. The registry was an observational study, and patient management was conducted entirely at the physician’s discretion. Additional subgroup analyses for each cancer type were pre-specified in the protocol. The present subgroup analysis focused on the stomach cancer cohort of the Cancer-VTE Registry.

The ethics committee or institutional review board at each participating institution approved the study protocol. The study was conducted in accordance with the Declaration of Helsinki and the Ethical Guidelines for Medical Science Studies on Human Subjects by the Japanese Ministry of Education, Culture, Sports, Science and Technology and the Ministry of Health, Labour and Welfare. All patients provided written informed consent before confirmation of eligibility.

### Patients

The main inclusion criteria were as follows: patients aged ≥ 20 years with primary or recurrent stomach cancer, clinical stages II–IV [13], life expectancy ≥ 6 months after registration, Eastern Cooperative Oncology Group performance status (ECOG PS) of 0–2, planning to initiate cancer treatment (i.e., chemotherapy, molecular targeted therapy, immunotherapy, radiation therapy, surgery or concomitant multidisciplinary treatment), and who underwent venous ultrasonography or computed tomography (CT) angiography of the lower extremity within 2 months before registration for VTE screening, as recommended by Japanese guidelines [14], unless D-dimer concentration after cancer diagnosis was ≤ 1.2 μg/mL, which would indicate non-VTE [15]. Contrast CT or other imaging tests were used to confirm the presence of pulmonary embolism when suspected.

The main exclusion criteria were the presence of active double cancer and being judged as inappropriate for inclusion or difficult to follow-up.

### Study outcomes

Outcomes were assessed at baseline and during follow-up in this subgroup analysis. Outcomes assessed at baseline were the prevalence of VTE and its risk factors. Outcomes assessed at follow-up were the incidence of symptomatic VTE; incidence of incidental (asymptomatic) VTE requiring treatment; combined incidence of these two types of VTE events (composite VTE); incidence of bleeding events (major or clinically relevant non-major bleeding); incidence of cerebral infarction/transient ischemic attack (TIA)/systemic embolic events (SEE); incidence of all-cause death; and analysis of risk factors for composite VTE and all-cause death.

### Statistical analysis

Details of the statistical analysis have been previously reported [10, 11]. Briefly, the planned sample size was 2,000 patients diagnosed with stomach cancer based on estimates for the Japanese population [16]. Frequency (n [%]) tables were developed for categorical variables, and summary statistics (mean, standard deviation, and median) were calculated for continuous variables.

Time-to-event rates were calculated using the cumulative incidence function for each event of interest at 365 days, and other survival time-to-event analyses and incidence of events were evaluated from 0 to 455 days. For analyses of intergroup differences, p values were calculated using either the Gray test (for VTE, bleeding, and cerebral infarction/TIA/SEE) or the log-rank test (for all-cause death).

Univariable and multivariable logistic regression analyses were conducted to investigate factors associated with VTE prevalence at baseline. Univariable and multivariable regression analyses were also conducted to investigate factors associated with the occurrence of composite VTE during the follow-up period using the Fine and Gray models (with all-cause death as a competing event) and to investigate factors associated with all-cause death during the follow-up period using the Cox proportional hazards model. The explanatory variables for the multivariable analysis were selected from univariable explanatory variables, considering the following two points: clinical importance and clearly correlated factors from a clinical perspective (e.g., cancer stage, lymph node metastasis, and distant metastasis are predicted to be correlated among these factors; therefore, only stage was selected). Adjustment factors for VTE risk at baseline were sex, age, BMI, creatinine clearance (CrCL), bed rest for 4 days or more, history of VTE, clinical stage, ECOG PS, occurrence of tumor, predominant histological type, platelet count, hemoglobin, white blood cell count, and D-dimer at baseline. Risk factors of composite VTE during the follow-up period were BMI, VTE at baseline, clinical stage, predominant histological type, and cancer therapy. Risk factors of all-cause death were sex, age, VTE at baseline, clinical stage, and ECOG PS.

Two-sided p < 0.05 was considered statistically significant. The statistical software used for these analyses was SAS version 9.4 (SAS Institute Inc., Cary, NC, USA).

## Results

### Patient characteristics

Of the 1,896 patients with stomach cancer in the Cancer-VTE Registry (N = 9,630), 131 (6.9%) patients had VTE at baseline, and 1,765 (93.1%) did not have VTE at baseline (Table 1). Overall, most patients were male (67.9%), the mean age was > 70 years. The most common predominant histology was the intestinal type (72.7%). Approximately 44.0% of patients had stage II disease, 73.6% had an ECOG PS of 0, and 23.7% had a D-dimer level > 1.2 μg/mL.

**Table 1:** Patient characteristics at baseline

	Total N = 1,896 (100.0)	With VTE n = 131 (6.9)	Without VTE n = 1,765 (93.1)
Male sex	1,287 (67.9)	60 (45.8)	1,227 (69.5)
Age, mean (SD)	70.0 ± 10.7	74.8 ± 9.0	69.6 ± 10.8
≥ 65 years	1,413 (74.5)	120 (91.6)	1,293 (73.3)
BMI, mean (SD)	22.19 ± 3.49	21.81 ± 3.18	22.22 ± 3.51
≥ 25	364 (19.2)	17 (13.0)	347 (19.7)
CrCL, mL/min, mean (SD)	70 ± 25	61 ± 22	71 ± 25
≤ 50	364 (19.2)	38 (29.0)	326 (18.5)
Clinical stage			
II	835 (44.0)	37 (28.2)	798 (45.2)
III	682 (36.0)	44 (33.6)	638 (36.1)
IV	379 (20.0)	50 (38.2)	329 (18.6)
ECOG PS			
0	1,396 (73.6)	75 (57.3)	1,321 (74.8)
1	420 (22.2)	40 (30.5)	380 (21.5)
2	80 (4.2)	16 (12.2)	64 (3.6)
Primary cancer	1,852 (97.7)	128 (97.7)	1,724 (97.7)
Recurrent cancer	44 (2.3)	3 (2.3)	41 (2.3)
With lymph node metastasis	1,261 (66.5)	104 (79.4)	1,157 (65.6)
With distant metastasis	367 (19.4)	51 (38.9)	316 (17.9)
Predominant histological type			
Intestinal type^a^	1,378 (72.7)	100 (76.3)	1,278 (72.4)
Diffuse type^b^	461 (24.3)	27 (20.6)	434 (24.6)
Others^c^	54 (2.8)	4 (3.1)	50 (2.8)
Platelet count, 10^9^/L, mean (SD)	274 ± 92	281 ± 91	273 ± 93
≥ 350	327 (17.2)	28 (21.4)	299 (16.9)
Hb, g/dL, mean (SD)	12.0 ± 2.4	10.8 ± 2.2	12.1 ± 2.4
< 10	389 (20.5)	43 (32.8)	346 (19.6)
WBC count, 10^9^/L, mean (SD)	6.51 ± 2.16	6.73 ± 2.22	6.49 ± 2.16
> 11	63 (3.3)	4 (3.1)	59 (3.3)
D-dimer, μg/mL, mean (SD)	1.63 ± 3.90	5.47 ± 10.24	1.33 ± 2.63
> 1.2	450 (23.7)	104 (79.4)	346 (19.6)
DOAC or warfarin use^d^	104 (5.5)	39 (29.8)	65 (3.7)

^a^Composite of papillary adenocarcinoma, tubular adenocarcinoma (well and moderately differentiated), and poorly differentiated adenocarcinoma (solid type)

^b^Composite of poorly differentiated adenocarcinoma (non-solid type), signet-ring cell carcinoma, and mucinous adenocarcinoma

^c^Composite of special type and others

^d^Oral anticoagulant treatment that started before enrollment

*BMI* body mass index, *CrCL* creatinine clearance, *DOAC* direct-acting oral anticoagulant, *ECOG PS* Eastern Cooperative Oncology Group performance status, *Hb* hemoglobin, *SD* standard deviation*, VTE* venous thromboembolism, *WBC* white blood cell

### Study outcomes

#### VTE at baseline

Table 2 shows the VTE details at baseline. Most VTE was asymptomatic, distal DVT. Univariable and multivariable analyses of factors correlated with VTE prevalence at baseline are shown in Online Resource 1. VTE at baseline was frequently observed in female patients, elderly patients, history of VTE, and those with poor ECOG PS, lymph node metastasis, distant metastasis, clinical stage IV, high D-dimer, low hemoglobin, or low CrCL. The predominant histological type was not associated with VTE at baseline. The independent risk factors for VTE prevalence at baseline were female sex, older age (≥ 65 years), history of VTE, and D-dimer > 1.2 µg/mL.

**Table 2:** Details of VTE found at baseline (N = 131)

	Total	Symptomatic VTE	Asymptomatic VTE
All VTE	131 (100.0)	5 (3.8)	126 (96.2)
PE (with/without DVT)	10 (7.6)	1 (0.8)	9 (6.9)
DVT (with/without PE)	127 (96.9)	4 (3.1)	123 (93.9)
Proximal DVT	17 (13.0)	2 (1.5)	15 (11.5)
Distal DVT	110 (84.0)	2 (1.5)	108 (82.4)

*DVT* deep vein thrombosis, *PE* pulmonary embolism, *VTE* venous thromboembolism

#### Incidence of events during follow-up

The mean follow-up duration was 346.4 days. During the follow-up period, symptomatic VTE was found in 0.3%, incidental VTE requiring treatment in 1.1%, composite VTE in 1.4%, bleeding in 1.6%, cerebral infarction/TIA/SEE in 0.7%, and all-cause death in 15.0% of patients (Table 3). The incidence of event components according to VTE at baseline is summarized in Online Resource 2.

**Table 3:** Incidence of events during the follow-up period

Event	All patients (N = 1,896)		With VTE at baseline (n = 131)		Without VTE at baseline (n = 1,765)	
	Patients with events, n	Incidence rate (95% CI)	Patients with events, n	Incidence rate (95% CI)	Patients with events, n	Incidence rate (95% CI)
Symptomatic VTE	6	0.3 (0.1–0.7)	0	0.0 (0.0–2.8)	6	0.3 (0.1–0.7)
Incidental VTE requiring treatment	20	1.1 (0.6–1.6)	3	2.3 (0.5–6.5)	17	1.0 (0.6–1.5)
Composite VTE^a^	26	1.4 (0.9–2.0)	3	2.3 (0.5–6.5)	23	1.3 (0.8–1.9)
Bleeding^b^	30	1.6 (1.1–2.3)	10	7.6 (3.7–13.6)	20	1.1 (0.7–1.7)
Cerebral infarction /TIA/SEE	13	0.7 (0.4–1.2)	0	0.0 (0.0–2.8)	13	0.7 (0.4–1.3)
All-cause death	285	15.0 (13.5–16.7)	46	35.1 (27.0–43.9)	239	13.5 (12.0–15.2)

^a^A composite of symptomatic VTE events and incidental VTE events requiring treatment

^b^Included major bleeding and clinically relevant non-major bleeding events

*CI* confidence interval, *SEE* systemic embolic event, *TIA* transient ischemic attack, *VTE* venous thromboembolism

**Fig. 1:**
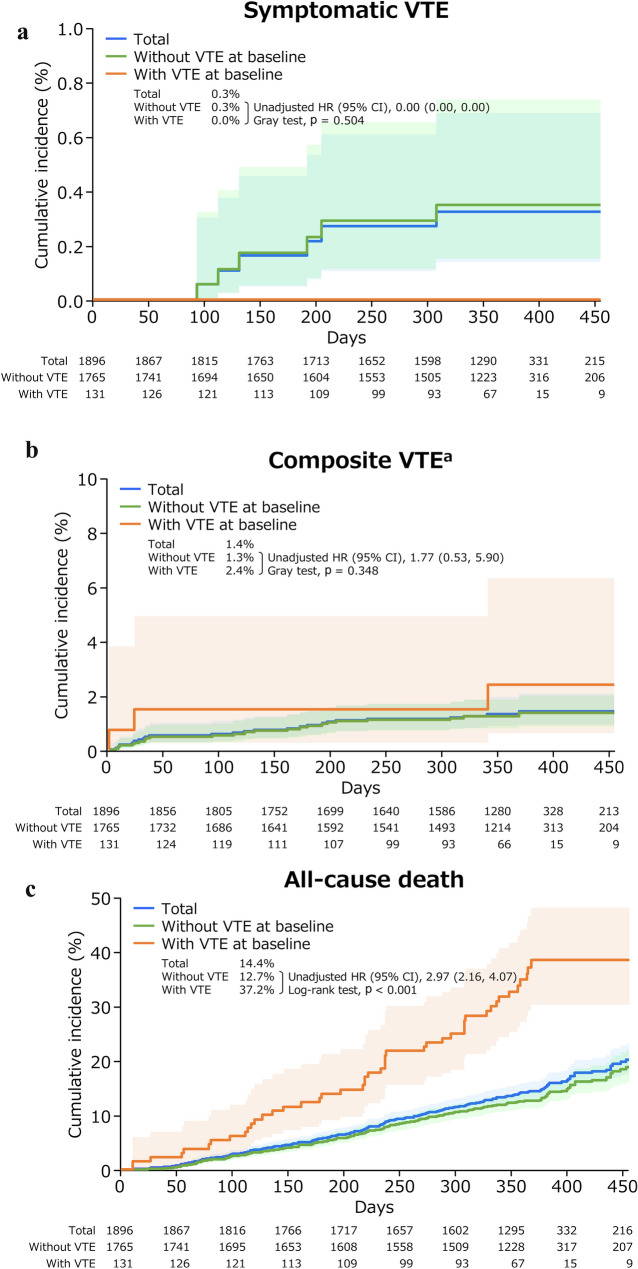
Cumulative incidence of events (time-to-event analysis). **a** Symptomatic VTE, **b** composite VTE, and **c** all-cause death. P values were calculated using the Gray test (**a**, **b**) or the log-rank test (**c**). Lightly shaded areas represent 95% CIs. ^a^A composite of symptomatic VTE events and incidental VTE events requiring treatment. *CI* confidence interval, *HR* hazard ratio, *VTE* venous thromboembolism

The 1-year cumulative incidences of each event are shown in Fig. 1 and Online Resource 3. The incidences of symptomatic VTE and cerebral infarction/TIA/SEE were low overall, and none of these events occurred among patients with VTE at baseline. Compared with patients without VTE at baseline, those with VTE had a higher risk of composite VTE (1-year cumulative incidence 2.4% vs 1.3%; unadjusted hazard ratio [HR] 1.77 [95% confidence interval {CI} 0.53–5.90]; p = 0.348 [Gray test]), all-cause death (37.2% vs 12.7%; 2.97 [2.16–4.07]; p < 0.001 [log-rank test]), and bleeding events (7.7% vs 1.1%; 7.03 [3.29–15.03]; p < 0.001 [Gray test]).

#### Event occurrence by subgroup

Table 4 summarizes the incidence of composite VTE during the follow-up period by subgroup according to disease characteristics. By predominant histological type, patients with diffuse histology had a higher incidence of composite VTE than those with intestinal histology. The incidence of composite VTE increased with cancer progression (i.e., clinical stage IV vs II and III) and poor PS (i.e., ECOG PS of 1 and 2). By cancer treatment type, patients who received chemotherapy had a higher incidence of composite VTE than those who underwent surgery (1.7 vs 1.1%). Among patients undergoing chemotherapy, those undergoing chemotherapy by intravenous injection had a higher incidence of composite VTE (2.4%).

**Table 4:** Incidence of composite VTE during the follow-up period by disease characteristics

	All	Composite VTE	
	N (%)	n (%)	95% CI
Total	1,896 (100.0)	26 (1.4)	0.9–2.0
Clinical stage			
II	835 (44.0)	10 (1.2)	0.6–2.2
III	682 (36.0)	8 (1.2)	0.5–2.3
IV	379 (20.0)	8 (2.1)	0.9–4.1
ECOG PS			
0	1,396 (73.6)	17 (1.2)	0.7–1.9
1	420 (22.2)	7 (1.7)	0.7–3.4
2	80 (4.2)	2 (2.5)	0.3–8.7
Predominant histological type			
Intestinal type^a^	1,378 (72.7)	16 (1.2)	0.7–1.9
Diffuse type^b^	461 (24.3)	10 (2.2)	1.0–4.0
Others^c^	54 (2.8)	0 (0.0)	0.0–6.6
Cancer therapy			
No	44 (2.3)	0 (0.0)	0.0–8.0
Surgery	1,568 (82.7)	17 (1.1)	0.6–1.7
(Surgery alone)	631 (33.3)	5 (0.8)	0.3–1.8
Chemotherapy	1,211 (63.9)	21 (1.7)	1.1–2.6
(Intravenous injection)	823 (43.4)	20 (2.4)	1.5–3.7
Radiotherapy	25 (1.3)	0 (0.0)	0.0–13.7

^a^Composite of papillary adenocarcinoma, tubular adenocarcinoma (well and moderately differentiated), and poorly differentiated adenocarcinoma (solid type)

^b^Composite of poorly differentiated adenocarcinoma (non-solid type), signet-ring cell carcinoma, and mucinous adenocarcinoma

^c^Composite of special type and others

*CI* confidence interval, *ECOG PS* Eastern Cooperative Oncology Group performance status, *VTE* venous thromboembolism

#### Risk factors for composite VTE

Table 5 shows the univariable and multivariable analyses of factors correlated with the incidence of composite VTE during the 1-year follow-up period. Multivariable analysis revealed that BMI of ≥ 25 kg/m^2^ (HR 2.90; 95% CI 1.23–6.87; p = 0.015) was a significant factor. Other factors, including BMI of < 18.5 kg/m^2^, prevalence of VTE at baseline, clinical stage, predominant histological type, and treatment (chemotherapy by intravenous injection) had higher HRs of > 1 but were not statistically significantly associated with composite VTE during the follow-up period. Online Resource 4 shows univariable analysis of variables that were not considered explanatory variables in the multivariable analysis.

**Table 5:** Univariable and multivariable analysis of risk factors for composite VTE during the follow-up period

Items		N	Events, n (%)	Univariable			Multivariable		
				HR	95% CI	p value	HR	95% CI	p value
BMI, kg/m^2^	≥ 25	364	9 (2.5)	2.89	1.20–6.95	0.018	2.90	1.23–6.87	0.015
	18.5 to < 25	1,282	11 (0.9)	Ref	–	–	Ref	–	–
	< 18.5	249	6 (2.4)	2.85	1.06–7.72	0.039	2.39	0.76–7.50	0.134
VTE at baseline	No	1,765	23 (1.3)	Ref	–	–	Ref	–	–
	Yes	131	3 (2.3)	1.77	0.53–5.90	0.352	1.80	0.51–6.36	0.360
Clinical stage	II/III	1,517	18 (1.2)	Ref	–	–	Ref	–	–
	IV	379	8 (2.1)	1.79	0.78–4.11	0.170	1.21	0.43–3.41	0.721
Predominant histological type	Intestinal^a^ /others^b^	1,432	16 (1.1)	Ref	–	–	Ref	–	–
	Diffuse type^c^	461	10 (2.2)	1.95	0.88–4.29	0.098	1.81	0.80–4.07	0.152
Cancer therapy	Other^d^	1,077	10 (0.9)	Ref	–	–	Ref	–	–
	Chemotherapy by intravenous injection^e^	819	16 (2.0)	2.09	0.95–4.59	0.068	1.70	0.72–4.05	0.230

^a^Composite of papillary adenocarcinoma, tubular adenocarcinoma (well and moderately differentiated), and poorly differentiated adenocarcinoma (solid type)

^b^Composite of special type and others

^c^Composite of poorly differentiated adenocarcinoma (non-solid type), signet-ring cell carcinoma, and mucinous adenocarcinoma

^d^Other than chemotherapy by intravenous injection prior to the occurrence of the composite VTE event

^e^Cancer therapy prior to the occurrence of the composite VTE event

The multivariable analysis used all-cause death as a competing event, and variables listed in this table as explanatory variables

*BMI* body mass index, *CI* confidence interval, *HR* hazard ratio, *Ref* reference, *VTE* venous thromboembolism

#### Risk factors for all-cause death

Univariable and multivariable analysis revealed that significant risk factors for all-cause death during the follow-up period were older age, VTE prevalence at baseline, advanced clinical cancer stage (III and IV), and poor ECOG PS (1 and 2) (Table 6).

**Table 6:** Univariable and multivariable analysis of risk factors for all-cause death during the follow-up period

Items		N	Events, n (%)	Univariable			Multivariable		
				HR	95% CI	p value	HR	95% CI	p value
Sex	Male	1,287	182 (14.1)	Ref	–	–	Ref	–	–
	Female	609	103 (16.9)	1.20	0.94–1.53	0.139	1.08	0.85–1.38	0.520
Age, years	< 65	483	51 (10.6)	Ref	–	–	Ref	–	–
	≥ 65	1,413	234 (16.6)	1.66	1.23–2.25	0.001	1.41	1.03–1.91	0.031
VTE at baseline	No	1,765	239 (13.5)	Ref	–	–	Ref	–	–
	Yes	131	46 (35.1)	2.97	2.16–4.07	< 0.001	1.67	1.21–2.32	0.002
Clinical stage	II	835	61 (7.3)	Ref	–	–	Ref	–	–
	III	682	73 (10.7)	1.52	1.08–2.13	0.016	1.43	1.02–2.01	0.039
	IV	379	151 (39.8)	6.87	5.10–9.25	< 0.001	5.63	4.14–7.66	< 0.001
ECOG PS	0	1,396	148 (10.6)	Ref	–	–	Ref	–	–
	1	420	115 (27.4)	2.88	2.25–3.67	< 0.001	1.89	1.47–2.43	< 0.001
	2	80	22 (27.5)	3.32	2.12–5.20	< 0.001	2.77	1.75–4.37	< 0.001

The multivariable analysis used variables listed in this table as explanatory variables

*CI* confidence interval, *ECOG PS* eastern cooperative oncology group performance status, *HR* hazard ratio, *Ref* reference, *VTE* venous thromboembolism

## Discussion

Given the lack of comprehensive, reliable data on the incidence of VTE in patients with stomach cancer in Japan, this was the first prospective, nationwide, observational study to collect and analyze data on VTE incidence among Japanese patients with stomach cancer in a real-world setting. This pre-specified subgroup analysis from the Cancer-VTE Registry examined the incidence of VTE among those undergoing VTE screening before initiating any cancer treatment and identified risk factors of VTE in Japanese patients with stomach cancer during a 1-year follow-up period.

Our previous reports from the overall Cancer-VTE Registry clarified that the overall VTE prevalence at baseline screening was 5.9% among the six major solid tumor groups, including 5.5% with asymptomatic VTE and 0.3% with symptomatic VTE [11, 12]. In the present analysis, 6.9% of patients with stomach cancer had VTE at baseline, and stomach cancer had the second highest incidence of VTE at baseline (6.9%) after pancreatic cancer (8.5%) [11, 12], indicating that the incidence of VTE was not negligible in patients with stomach cancer in Japan.

Among patients with stomach cancer, female sex, age ≥ 65 years, history of VTE, and high D-dimer level (> 1.2 μg/mL) were identified as risk factors associated with VTE prevalence at baseline. These results are almost concordant with previous studies [17, 18]. Of note, patients with D-dimer > 1.2 µg/mL at the time of cancer diagnosis had an approximately 20-fold risk of VTE. A stepwise screening procedure that measures D-dimer at least at the time of cancer diagnosis and incorporates imaging tests for suspected VTE at high levels might be an efficient and effective strategy for revealing VTE, including asymptomatic VTE. The American Society of Clinical Oncology has published a guideline on VTE in cancer patients [19], but there is no mention of VTE in the Japanese Gastric Cancer Treatment Guidelines [20]. The results of this study, which clarify the current situation in the Japanese population, may help inform future guideline updates.

During the follow-up period, the cumulative incidences of symptomatic and composite VTE were 0.3% and 1.4%, respectively, which were similar to, or even lower than, the incidence reported in previous Asian studies. A study of Asian patients with advanced inoperable stomach cancer treated with systemic chemotherapy reported a 1-year cumulative incidence of 3.5%, and patients concurrently diagnosed with cancer and VTE had worse prognoses than those without VTE [21]. A recent study of patients with metastatic stomach cancer in Japan reported that the incidence of VTE at the start of, or during chemotherapy was 18% [22]. A possible explanation for the lower incidence of VTE in this study compared with these reports is that this study recruited more surgical patients (0% in these previous reports; 82.7% in the present study) [21, 22]. A previous study, which included Japanese patients undergoing laparoscopic surgery for gastrointestinal malignancy without chemotherapy, also reported a similarly low incidence of VTE as in the present study (2.6% in the overall population; 1.2% in the population receiving low molecular weight heparin) [23]. Principally, surgery is indicated for localized disease and chemotherapy alone for patients with distant metastasis. Advanced stage and chemotherapy have been associated with an increased VTE risk [24]. In fact, in this study, VTE occurred more frequently in patients undergoing chemotherapy than those undergoing surgical resection alone. Moreover, the present results were generally consistent with a study of surgical cases in Japan [17, 25].

The incidence of composite VTE increased in patients with BMI ≥ 25 kg/m^2^, which was compatible with a previous report of Japanese patients with stomach cancer [26]. Other factors such as BMI < 18.5 kg/m^2^, VTE prevalence at baseline, clinical stage, predominant histological type, and treatment (chemotherapy by intravenous injection) had HRs ≥ 1 but were not statistically significant and were not independent risk factors. However, the low number of events may have limited this analysis.

An unexpected finding was that patients receiving chemotherapy by intravenous injection did not show statistically higher HR for VTE (1.70; 95% CI 0.72–4.05; p = 0.230) than those not receiving chemotherapy. Although many previous studies showed an increased risk of VTE with anti-cancer drug use [24, 27], most studies included patients with metastatic disease. In the present study, the disease stage of patients receiving chemotherapy was not limited to stage IV only. In fact, this study included patients with disease stage II or III, which may have affected the results negatively.

The difference in incidence of VTE events during the follow-up period did not reach statistical significance between patients with and without VTE prevalence at baseline. However, the cumulative incidence of all-cause death during the follow-up period was more than double in patients with VTE at baseline (37.2%) than those without VTE at baseline (12.7%; p < 0.001). Additionally, VTE at baseline was a risk factor of all-cause death (adjusted HR 1.67; 95% CI 1.21–2.32; p = 0.002). At the time of cancer diagnosis, diagnosis of the presence or absence of VTE, including asymptomatic cases, as a prognostic indicator would be useful in planning individualized cancer treatment for each patient. These findings highlight the importance of measuring D-dimer and screening VTE early after cancer diagnosis in patients with stomach cancer.

Theoretically, patients with VTE at baseline should have been at an increased risk of VTE recurrence during cancer treatment, which was not the case in our study. Further, although not statistically significant, there were no occurrences of cerebral infarction/TIA/SEE in patients with VTE at baseline. This may be attributable to adequate prophylaxis, such as the use of anticoagulants (oral anticoagulant use: 29.8% with VTE at baseline, 3.7% without VTE at baseline). Conversely, patients with VTE at baseline had a higher risk of bleeding (unadjusted HR 7.03; 95% CI 3.29–15.03; p < 0.001) than those without VTE at baseline. Thus, use of anticoagulants for VTE should be determined considering these risks and benefits to prevent VTE development during cancer treatment.

This study had some limitations, including the short follow-up duration. The observational design also limits the data that can be obtained. Moreover, the VTE events observed were lower than expected, resulting in statistically non-significant differences in several analyses. Furthermore, the Cancer-VTE Registry included only Japanese patients, limiting the generalizability of the findings to other Asian populations. Chemotherapy in this study included both systemic chemotherapy for advanced cancer and adjuvant chemotherapy for early-stage cancer. Unfortunately, it was not possible to study only chemotherapy for advanced cancer because we did not collect detailed data to this end. The Cancer-VTE Registry was also limited in terms of life expectancy and PS in addition to stage, which limited our ability to compare the results with previous studies that matched patients’ backgrounds. Finally, asymptomatic VTE cannot be diagnosed definitively without imaging studies. However, in this study, VTE testing during the observation period followed the routine practice of the participating centers. We did not specify a protocol visit, which may have been another reason for the low frequency of VTE occurrence.

## Conclusions

In Japanese patients with stage II–IV stomach cancer undergoing cancer treatment, VTE prevalence at the time of cancer diagnosis was not negligible (6.9%) but mainly comprised asymptomatic/distal DVT. Risk factors of VTE prevalence at baseline were female sex, age ≥ 65 years, VTE history, and D-dimer > 1.2 μg/mL, with high D-dimer being the highest risk factor. Especially, patients with D-dimer > 1.2 µg/mL at the time of cancer diagnosis had an approximately 20-fold risk of VTE. Moreover, VTE at baseline was a significant indicator of poor prognosis. VTE screening by D-dimer before starting cancer treatment is advisable even for asymptomatic patients, regardless of whether the patient is undergoing surgery or receiving chemotherapy.

## Supplementary Information

Below is the link to the electronic supplementary material.

Supplementary file1 (DOCX 176 KB)

## Data Availability

The anonymized data underlying the results presented in this manuscript may be made available to researchers upon submission of a reasonable request to the corresponding author. The decision to disclose the data will be made by the corresponding author and the funder, Daiichi Sankyo Co., Ltd. The data disclosure can be requested for 36 months from the article publication.
