# Supplementary material for: Incidence and risk factors for venous thromboembolism in the Cancer-VTE Registry stomach cancer subcohort
Source: Gastric Cancer. 2023 Apr 1;26(4):493–503. doi: 10.1007/s10120-023-01378-1 (PMC10284943; doi:10.1007/s10120-023-01378-1)
Supplement: Supplementary file 1 — Supplementary file1 (DOCX 176 KB) [file 10120_2023_1378_MOESM1_ESM.docx]

**Incidence and risk factors for venous thromboembolism in the Cancer-VTE Registry stomach cancer subcohort**

**Journal: Gastric Cancer**

Takaki Yoshikawa^1^, Takeshi Sano^2^, Masanori Terashima^3^, Kensei Yamaguchi^4^, Etsuro Bando^3^, Ryohei Kawabata^5,6^, Hiroshi Yabusaki^7^, Hisashi Shinohara^8^, Mari S Oba^9,10^, Tetsuya Kimura^11^, Atsushi Takita^12^, Mitsuru Sasako^13^

1. Department of Gastric Surgery, National Cancer Center Hospital, 5-1-1 Tsukiji, Chuo-ku, Tokyo 104-0045, Japan
2. Department of Gastroenterological Surgery, Cancer Institute Hospital of the Japanese Foundation for Cancer Research, Tokyo, Japan
3. Division of Gastric Surgery, Shizuoka Cancer Center, Shizuoka, Japan
4. Department of Gastroenterological Chemotherapy, Cancer Institute Hospital of the Japanese Foundation for Cancer Research, Tokyo, Japan
5. Department of Surgery, Osaka Rosai Hospital, Sakai, Japan
6. Department of Surgery, Sakai City Medical Center, Sakai, Japan
7. Department of Gastroenterological Surgery, Niigata Cancer Center Hospital, Niigata, Japan
8. Department of Gastroenterological Surgery, Division of Upper GI, Hyogo Medical University, Nishinomiya, Japan
9. Department of Medical Statistics, Toho University, Tokyo, Japan
10. Department of Clinical Data Science, Clinical Research & Education Promotion Division, National Center of Neurology and Psychiatry, Tokyo, Japan
11. Primary Medical Science Department, Daiichi Sankyo Co., Ltd., Tokyo, Japan
12. Data Intelligence Department, Daiichi Sankyo Co., Ltd., Tokyo, Japan
13. Department of Surgery, Yodogawa Christian Hospital, Osaka, Japan

## Corresponding author

Takaki Yoshikawa

Department of Gastric Surgery, National Cancer Center Hospital

Postal address: 5-1-1 Tsukiji, Chuo-ku, Tokyo 104-0045, Japan

Tel: +81-3-3542-2511

E-mail: tayoshik@ncc.go.jp

**Online Resource 1**. Univariable and multivariable analysis of factors correlated with VTE prevalence at baseline

| **Factor** |  | **N** | **Events,**  **n (%)** | **Univariable** | | | **Multivariable** | | |
| --- | --- | --- | --- | --- | --- | --- | --- | --- | --- |
|  |  |  |  | OR | 95% CI | p value | OR | 95% CI | p value |
| Sex | Male | 1,287 | 60 (4.7) | Ref | - | - | Ref | - | - |
|  | Female | 609 | 71 (11.7) | 2.70 | 1.89–3.86 | < 0.001 | 2.55 | 1.64–3.95 | < 0.001 |
| Age, years | < 65 | 483 | 11 (2.3) | Ref | - | - | Ref | - | - |
|  | ≥ 65 | 1,413 | 120 (8.5) | 3.98 | 2.13–7.45 | < 0.001 | 3.35 | 1.51–7.47 | 0.003 |
| BMI | ≥ 25 | 364 | 17 (4.7) | 0.61 | 0.36–1.04 | 0.069 | 0.58 | 0.31–1.10 | 0.094 |
|  | 18.5 to < 25 | 1,282 | 95 (7.4) | Ref | - | - | Ref | - | - |
|  | < 18.5 | 249 | 19 (7.6) | 1.03 | 0.62–1.72 | 0.904 | 0.73 | 0.39–1.37 | 0.326 |
| CrCL, mL/min | > 50 | 1,486 | 87 (5.9) | Ref | - | - | Ref | - | - |
|  | ≤ 50 | 364 | 38 (10.4) | 1.87 | 1.26–2.80 | 0.002 | 0.67 | 0.41–1.12 | 0.126 |
| Bed rest for 4 days or more | No | 1,872 | 128 (6.8) | Ref | - | - | Ref | - | - |
|  | Yes | 24 | 3 (12.5) | 1.95 | 0.57–6.61 | 0.286 | 0.38 | 0.05–2.84 | 0.347 |
| History of VTE | No | 1,878 | 121 (6.4) | Ref | - | - | Ref | - | - |
|  | Yes | 18 | 10 (55.6) | 18.15 | 7.04–46.83 | < 0.001 | 24.97 | 7.52–82.88 | < 0.001 |
| Clinical stage | II/III | 1,517 | 81 (5.3) | Ref | - | - | Ref | - | - |
|  | IV | 379 | 50 (13.2) | 2.70 | 1.86–3.91 | < 0.001 | 1.50 | 0.92–2.43 | 0.106 |
| ECOG PS | 0 | 1,396 | 75 (5.4) | Ref | - | - | Ref | - | - |
|  | 1 | 420 | 40 (9.5) | 1.85 | 1.24–2.77 | 0.003 | 1.05 | 0.64–1.72 | 0.859 |
|  | 2 | 80 | 16 (20.0) | 4.40 | 2.43–7.99 | < 0.001 | 1.77 | 0.87–3.60 | 0.116 |
| Occurrence of tumor | Primary | 1,852 | 128 (6.9) | Ref | - | - | Ref | - | - |
|  | Recurrence | 44 | 3 (6.8) | 0.99 | 0.30–3.23 | 0.981 | 0.33 | 0.06–1.83 | 0.204 |
| Lymph node metastasis | No | 635 | 27 (4.3) | Ref | - | - | - | - | - |
|  | Yes | 1,261 | 104 (8.2) | 2.02 | 1.31–3.13 | 0.002 | - | - | - |
| Distant metastasis | No | 1,529 | 80 (5.2) | Ref | - | - | - | - | - |
|  | Yes | 367 | 51 (13.9) | 2.92 | 2.02–4.24 | < 0.001 | - | - | - |
| Predominant histological type | Intestinal^a^/others^b^ | 1,432 | 104 (7.3) | Ref | - | - | Ref | - | - |
|  | Diffuse^c^ | 461 | 27 (5.9) | 0.79 | 0.51–1.23 | 0.302 | 0.88 | 0.51–1.52 | 0.655 |
| Platelet count, 10^9^/L | < 350 | 1,523 | 97 (6.4) | Ref | - | - | Ref | - | - |
|  | ≥ 350 | 327 | 28 (8.6) | 1.38 | 0.89–2.14 | 0.153 | 1.30 | 0.74–2.29 | 0.362 |
| Hb, g/dL | ≥ 10 | 1,461 | 82 (5.6) | Ref | - | - | Ref | - | - |
|  | < 10 | 389 | 43 (11.1) | 2.09 | 1.42–3.08 | < 0.001 | 0.81 | 0.48–1.35 | 0.421 |
| WBC count,  10^9^/L | ≤ 11 | 1,787 | 121 (6.8) | Ref | - | - | Ref | - | - |
|  | > 11 | 63 | 4 (6.3) | 0.93 | 0.33–2.61 | 0.896 | 0.63 | 0.20–2.05 | 0.446 |
| D-dimer, μg/mL | ≤ 1.2 | 1,196 | 17 (1.4) | Ref | - | - | Ref | - | - |
|  | > 1.2 | 450 | 104 (23.1) | 20.84 | 12.31–35.28 | < 0.001 | 18.92 | 10.42–34.36 | < 0.001 |

Adjustment factors for VTE risk at baseline were sex, age, BMI, CrCL, bed rest for 4 days or more, history of VTE, clinical stage, ECOG PS, occurrence of tumor, predominant histological type, platelet count, Hb, WBC count, and D-dimer at baseline.

^a^Composite of papillary adenocarcinoma, tubular adenocarcinoma (well and moderately differentiated), and poorly differentiated adenocarcinoma (solid type).

^b^Composite of special type and others.

^c^Composite of poorly differentiated adenocarcinoma (non-solid type), signet-ring cell carcinoma, and mucinous adenocarcinoma.

*BMI* body mass index, *CI* confidence interval, *CrCL* creatinine clearance, *ECOG PS* Eastern Cooperative Oncology Group performance status, *Hb* hemoglobin, *OR* odds ratio, *Ref* reference, *VTE* venous thromboembolism, *WBC* white blood cell

**Online Resource 2**. Event components during the follow-up period.

|  | **All (N = 1,896)** | | **With VTE at baseline**  **(N = 131)** | | **Without VTE at baseline**  **(N = 1,765)** | |  |
| --- | --- | --- | --- | --- | --- | --- | --- |
|  | n | Incidence rate, %  (95% CI) | *n* | Incidence rate, %  (95% CI) | *n* | Incidence rate, %  (95% CI) | |
| Symptomatic PE | 0 | 0.0  (0.0–0.2) | 0 | 0.0  (0.0–2.8) | 0 | 0.0  (0.0–0.2) | |
| Symptomatic DVT | 6 | 0.3  (0.1–0.7) | 0 | 0.0  (0.0–2.8) | 6 | 0.3  (0.1–0.7) | |
| Asymptomatic PE | 6 | 0.3  (0.1–0.7) | 0 | 0.0  (0.0–2.8) | 6 | 0.3  (0.1–0.7) | |
| Asymptomatic DVT | 15 | 0.8  (0.4–1.3) | 3 | 2.3  (0.5–6.5) | 12 | 0.7  (0.4–1.2) | |
| Major bleeding | 12 | 0.6  (0.3–1.1) | 5 | 3.8  (1.3–8.7) | 7 | 0.4  (0.2–0.8) | |
| Clinically relevant  non-major bleeding | 18 | 0.9  (0.6–1.5) | 5 | 3.8  (1.3–8.7) | 13 | 0.7  (0.4–1.3) | |
| Cerebral infarction | 11 | 0.6  (0.3–1.0) | 0 | 0.0  (0.0–2.8) | 11 | 0.6  (0.3–1.1) | |
| TIA | 2 | 0.1  (0.0–0.4) | 0 | 0.0  (0.0–2.8) | 2 | 0.1  (0.0–0.4) | |
| SEE | 0 | 0.0  (0.0–0.2) | 0 | 0.0  (0.0–2.8) | 0 | 0.0  (0.0–0.2) | |

*CI* confidence interval, *DVT* deep vein thrombosis, *PE* pulmonary embolism, *SEE* systemic embolic event, *TIA* transient ischemic attack, *VTE* venous thromboembolism

**(a)**

**
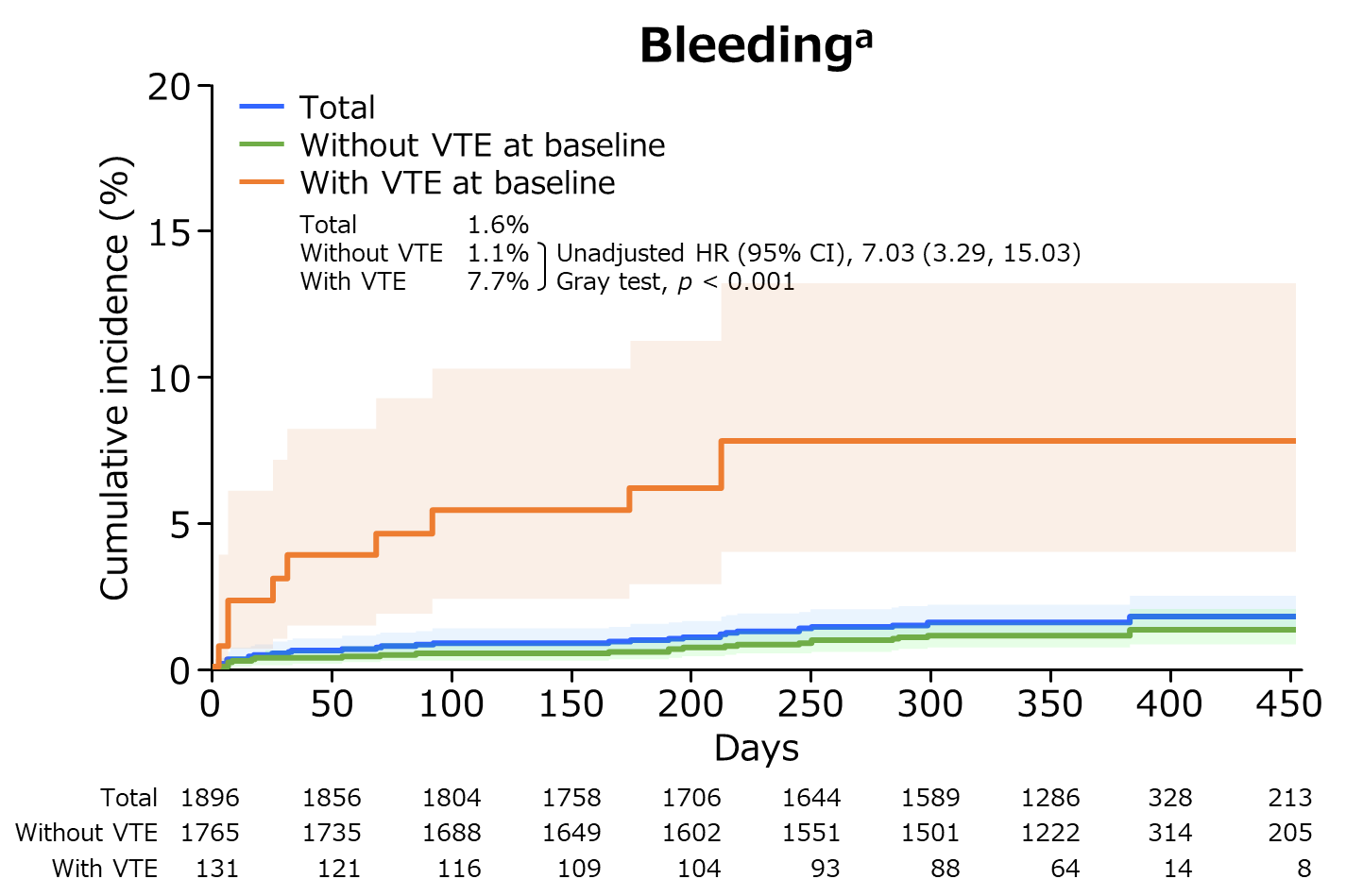
**

**(b)**

**
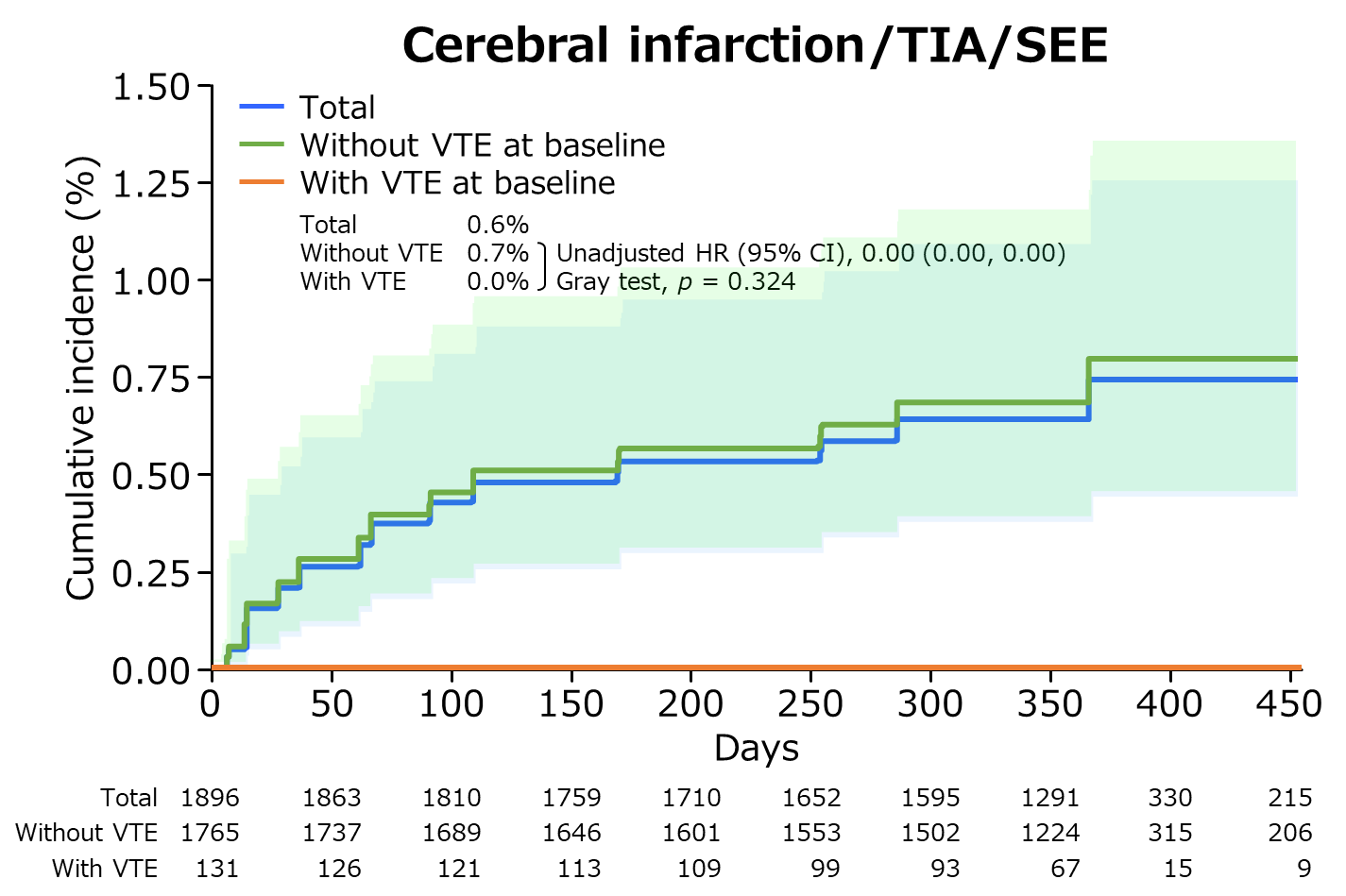
**

**Online Resource 3** Cumulative incidence of events (time-to-event analysis). (a) bleeding events, and (b) cerebral infarction/TIA/SEE.

The Gray test was used to calculate *p* values. Lightly shaded areas represent 95% CIs.

^a^Included major bleeding and clinically relevant non-major bleeding events.

*CI* confidence interval, *HR* hazard ratio, *SEE* systemic embolic event, *TIA* transient ischemic attack, *VTE* venous thromboembolism.

**Online Resource 4**. Univariable analysis of risk factors for composite VTE during the follow-up period

| **Factor** |  | **N** | **Events,**  **n (%)** | **Univariable** | | |
| --- | --- | --- | --- | --- | --- | --- |
|  |  |  |  | HR | 95% CI | p value |
| Sex | Male | 1,287 | 14 (1.1) | Ref | - | - |
|  | Female | 609 | 12 (2.0) | 1.84 | 0.85–3.97 | 0.123 |
| Age, years | < 65 | 483 | 8 (1.7) | Ref | - | - |
|  | ≥ 65 | 1,413 | 18 (1.3) | 0.77 | 0.34–1.78 | 0.545 |
| CrCL, mL/min | > 50 | 1,486 | 19 (1.3) | Ref | - | - |
|  | ≤ 50 | 364 | 2 (0.5) | 0.44 | 0.10–1.89 | 0.270 |
| Bed rest for 4 days or more | No | 1,872 | 26 (1.4) | Ref | - | - |
|  | Yes | 24 | 0 (0.0) | <0.01 | <0.01–<0.01 | <0.001 |
| History of VTE | No | 1,878 | 25 (1.3) | Ref | - | - |
|  | Yes | 18 | 1 (5.6) | 4.28 | 0.55–33.00 | 0.164 |
| ECOG PS | 0 | 1,396 | 17 (1.2) | Ref | - | - |
|  | 1 | 420 | 7 (1.7) | 1.37 | 0.57–3.29 | 0.480 |
|  | 2 | 80 | 2 (2.5) | 2.19 | 0.51–9.44 | 0.294 |
| Occurrence of tumor | Primary | 1,852 | 24 (1.3) | Ref | - | - |
|  | Recurrence | 44 | 2 (4.5) | 3.52 | 0.83–14.97 | 0.088 |
| Lymph node metastasis | No | 635 | 11 (1.7) | Ref | - | - |
|  | Yes | 1,261 | 15 (1.2) | 0.69 | 0.32–1.51 | 0.354 |
| Distant metastasis | No | 1,529 | 18 (1.2) | Ref | - | - |
|  | Yes | 367 | 8 (2.2) | 1.86 | 0.81–4.28 | 0.143 |
| Platelet count, 10^9^/L | < 350 | 1,523 | 19 (1.2) | Ref | - | - |
|  | ≥ 350 | 327 | 2 (0.6) | 0.49 | 0.11–2.10 | 0.336 |
| Hb, g/dL | ≥ 10 | 1,461 | 17 (1.2) | Ref | - | - |
|  | < 10 | 389 | 4 (1.0) | 0.89 | 0.30–2.65 | 0.836 |
| WBC count,  10^9^/L | ≤ 11 | 1,787 | 20 (1.1) | Ref | - | - |
|  | > 11 | 63 | 1 (1.6) | 1.46 | 0.20–10.93 | 0.711 |
| D-dimer, μg/mL | ≤ 1.2 | 1,196 | 14 (1.2) | Ref | - | - |
|  | > 1.2 | 450 | 10 (2.2) | 1.95 | 0.87–4.39 | 0.106 |

Variables presented in this table were not used in the multivariate analysis of risk factors for composite VTE during the follow-up period.

*CI* confidence interval, *CrCL* creatinine clearance, *ECOG PS* Eastern Cooperative Oncology Group performance status, *Hb* hemoglobin, *HR* hazard ratio, *Ref* reference, *VTE* venous thromboembolism, *WBC* white blood cell
